# Interpreting the impact of noncoding structural variation in neurodevelopmental disorders

**DOI:** 10.1038/s41436-020-00974-1

**Published:** 2020-09-25

**Authors:** Eva D’haene, Sarah Vergult

**Affiliations:** Center for Medical Genetics, Department of Biomolecular Medicine, Ghent University, Ghent, Belgium

**Keywords:** neurodevelopmental disorders, noncoding variation, structural variation, gene regulation, chromatin conformation

## Abstract

The emergence of novel sequencing technologies has greatly improved the identification of structural variation, revealing that a human genome harbors tens of thousands of structural variants (SVs). Since these SVs primarily impact noncoding DNA sequences, the next challenge is one of interpretation, not least to improve our understanding of human disease etiology. However, this task is severely complicated by the intricacy of the gene regulatory landscapes embedded within these noncoding regions, their incomplete annotation, as well as their dependence on the three-dimensional (3D) conformation of the genome. Also in the context of neurodevelopmental disorders (NDDs), reports of putatively causal, noncoding SVs are accumulating and understanding their impact on transcriptional regulation is presenting itself as the next step toward improved genetic diagnosis.

## INTRODUCTION

Structural variation (SV) represents the greatest source of genetic diversity in the human genome^1–3^. Copy-number variants (CNVs), such as deletions and duplications, as well as balanced genomic rearrangements, e.g., translocations and inversions, affect more base pairs than single-nucleotide variants (SNVs)^1–3^. CNVs, per definition, result in a gain or loss of DNA and can therefore affect gene dosage. Balanced rearrangements on the other hand, although not accompanied by dosage alterations, can have a significant impact on linear and 3D chromatin conformation.

Despite their large impact on genome structure, it remains a challenging task to comprehensively map all SVs in a human genome. Microarray technology, long the standard in clinical diagnostics to identify DNA gains and losses, neither allows the precise mapping of breakpoints, nor the detection of balanced rearrangements. The advent of next-generation sequencing technologies has improved SV discovery, although short-read genome sequencing (GS) approaches have trouble detecting SVs in repeat-rich regions. Therefore, the most comprehensive overview of human structural variation to date has been achieved through a combination of short-read GS and long-range sequencing technologies, identifying on average over 27,000 SVs per genome^2^. For an in-depth discussion of strategies and algorithms for SV detection we refer to other reviews^4,5^.

Given their size (per definition >50 bp), it is unsurprising that germline SVs can contribute greatly to congenital disease^6^. Both de novo and inherited SVs are frequently linked to the pathogenesis of neurodevelopmental disorders (NDDs)^6–10^. NDDs are a heterogeneous group of phenotypes in which normal development and functioning of the brain is disrupted, resulting in impairment of motor and behavior skills, speech, vision, hearing, cognition, etc. They include, among others, autism spectrum disorder (ASD), intellectual disability (ID), schizophrenia (SCZ), and developmental delay (DD). Moreover, these disorders are often syndromic, with patients also exhibiting a range of other, non-neurological comorbidities.

It has been estimated that gene dosage alterations caused by large CNVs are responsible for ~15% of NDD cases^11^. Apart from affecting the protein-coding portion of the genome, it has also been clearly established that SVs can cause disease through noncoding mechanisms, by altering the copy number or position of regulatory elements, or by reshuffling higher-order chromatin structures^12^. The overall contribution of such regulatory SV effects in disease etiology is still unclear. Yet, several experimentally validated examples, in particular in the context of limb development, have demonstrated their clinical importance and highlighted the diverse ways in which SVs can influence gene regulation^12^. As development of the brain, the most complex human organ, is tightly regulated, the impact of noncoding SVs should also be carefully considered in the context of neurodevelopmental disease. Although the literature contains multiple examples of noncoding SVs disrupting loci linked to NDD etiology, a comprehensive overview of these cases and the underlying, noncoding disease mechanisms is currently lacking. Therefore, this review aims to shed more light on the importance of noncoding SVs in NDD etiology by discussing (1) the noncoding functional elements involved in gene regulation during neurodevelopment, (2) the contribution of (noncoding) structural variation in NDDs, and (3) an extensive collection of reported NDD cases in which noncoding SVs appear to be at the root of the NDD phenotype.

## GENE REGULATION IN NEURODEVELOPMENT

Development of the human brain is a highly regulated process in which genes must be switched on in the right place at the right time. Dynamic, spatiotemporal gene expression programs orchestrate all stages of neurodevelopment, including neural stem cell proliferation, neuronal differentiation, and, ultimately, the migration and integration of postmitotic neurons^13^. Errors in the regulation of any of these processes could result in aberrant development and give rise to NDDs.

### The activity of noncoding functional elements regulates neurodevelopment

**Fig. 1:**
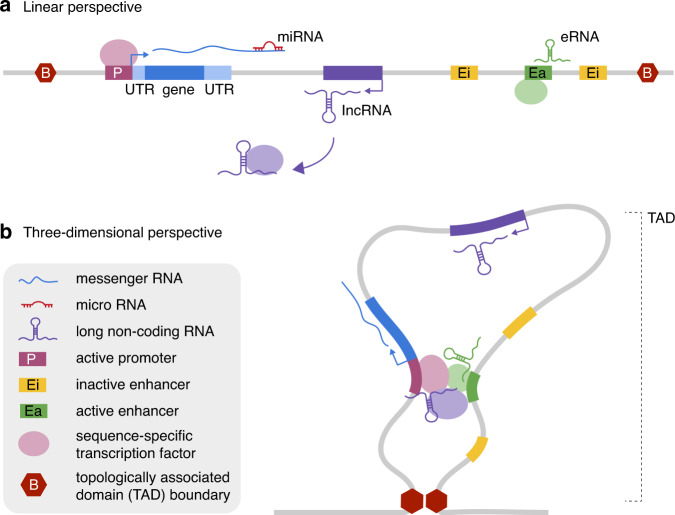
The gene regulatory landscape from a linear and three-dimensional perspective. (**a**) Genetic locus, illustrating the regulatory function of multiple noncoding elements. The region is delimited by topologically associated domain (TAD) boundaries on either side, each consisting of a cluster of CTCF binding sites. A protein-coding sequence is flanked by a promoter and 5’ and 3’ untranslated region (UTR). An intergenically transcribed long noncoding RNA (lncRNA) performs its regulatory function by acting as a scaffold for the binding of transcription factors (TFs). The activity of multiple enhancer elements in the locus is tissue- and even cell-type-dependent. (**b**) Via a loop extrusion mechanism, anchored by the CTCF-bound TAD boundaries, the functional elements in the locus are brought into close physical proximity, allowing interaction between the promoter and active enhancers and the assembly of the transcriptional machinery.

The regulatory machinery steering these transcriptional programs depends on the transcription of noncoding RNA (ncRNA) molecules, the activity of noncoding regulatory elements such as enhancers, and the 3D interaction between these noncoding regulatory sites and protein-coding target genes (Fig. 1).

### Noncoding RNAs

Transcription of noncoding sequences by RNA polymerase II is widespread in the human genome^14^. Both the process of transcription itself, as well as the resulting ncRNA molecules, can have a regulatory function. Small (21–25 nt) microRNAs (miRNAs) are primarily associated with gene repression, among others, via binding to the 3’ untranslated region (3’UTR) of genes (Fig. 1)^15^. Several examples highlight their relevance to NDD etiology. For instance, miR-137 is thought to perform a regulatory role during neurodevelopment and has been associated with SCZ and other neuropsychiatric disorders through genome-wide association studies (GWAS)^16^. Also, the overexpression of miRNAs on chromosome 21 and ensuing haploinsufficiency of their target genes is thought to contribute to ID in Down syndrome patients^17^.

However, the largest and most diverse group of ncRNA molecules is that of the long noncoding RNAs (lncRNAs). These are per definition longer than 200 nucleotides and include transcripts overlapping other genes (sense or antisense), intergenic transcripts, as well as enhancer RNAs (eRNAs) transcribed from active enhancer elements^18^. LncRNAs perform their regulatory activity via different mechanisms, both in *cis*, at the site of transcription, and in *trans*. They can influence gene regulation by acting as scaffolds, mediating the formation and sequence-specific binding of regulatory protein complexes, or decoys, sequestering and therefore inactivating transcription factors (TFs) and miRNAs (Fig. 1)^18^. However, as regulatory elements (both promoters and enhancers) initiate bidirectional transcription, it is possible that many antisense lncRNAs and eRNAs are by-products of this process and do not fulfill sequence-specific functions^19^. In these cases, the act of transcription itself or underlying regulatory element may still contribute to gene regulation^20^.

Although the function of many lncRNAs remains elusive and their functionality in some cases uncertain, several observations have suggested that they play an important role during neurodevelopment. Expression profiling by the GENCODE consortium showed that many lncRNAs are tissue-specific, with the largest group (~40%) being expressed specifically in the brain^21^. There are multiple examples of lncRNAs fulfilling a specific regulatory function in all stages of neurodevelopment and in neuronal plasticity^18^. The *Evf2* lncRNA, transcribed from a *Dlx5/6* enhancer, recruits the transcription factors Dlx and Mecp2 to *Dlx5/6* enhancer elements^22^. Moreover, it also influences chromatin topology to regulate *Dlx5/6* enhancer interactions by binding both activated and repressed target genes on chromosome 6 and regulating cohesin positioning^23^. *Pnky* is involved in neocortical development by regulating neuronal differentiation^24^. Although being transcribed divergently from the *Pou3f2* locus, it works via a *trans* mechanism. *Dlx1as* and *Six3os* both play a role in glial–neuronal lineage specification of neural stem cells^25^, while *Paupar* regulates olfactory bulb neurogenesis^26^. Kleaveland et al. even described a regulatory network in which a lncRNA (*Cyrano*), a circular RNA (*Cdr1as*), and two miRNAs (*miR-7* and *miR-671*) work together to regulate neuronal activity^27^.

As might be expected given their role in neurodevelopment, lncRNAs have also been implicated in NDDs, among others through GWAS^28^. Also, they were found to be enriched in CNVs identified in ASD patients (Alinejad-Rokny et al., unpublished data) and showed differential expression patterns in blood and brain tissue samples from ASD and major depressive disorder (MDD) patients^29–31^. However, it must be stressed that these disease associations are not conclusive evidence that the implicated lncRNAs play a role in disease etiology.

### Regulatory elements

Both proximal and distal noncoding regulatory sequences interact to fine tune protein expression levels. The former class, found adjacent to the protein-coding gene body, includes the promoter, which facilitates binding of RNA polymerase II and initiation of transcription, and the 3’UTR, harboring miRNA binding sites to mediate gene repression. Variation in the promoter as well as the 3’UTR of developmental genes has been linked to NDDs^32,33^. For example, several studies leveraged exome sequencing data to identify noncoding SNVs with a putative regulatory effect in 3’UTRs in patients with ASD, ID, and specific language impairment^34,35^.

Yet, the most abundant noncoding regulatory elements are enhancers, short DNA sequences that can activate gene expression by recruiting the transcriptional machinery (sequence-specific TFs, coactivators, and RNA polymerase II) to a target promoter in a stage- and tissue-specific manner^14^. Whether or not enhancers and other regulatory elements are active in certain tissues or at specific developmental stages is determined epigenetically, through DNA methylation and post-translational histone modifications (reviewed in^36,37^). Promoters are often regulated by multiple enhancers, which can display both overlapping and distinct spatiotemporal activity patterns. While enhancers with overlapping activities confer a level of redundancy that ensures a robust transcriptional output resistant to genetic variation^38^, those exhibiting differential activities determine the full spectrum of target gene activity. For example, tissue-specific enhancers are active in different subregions of the cerebral cortex, driving precise spatial expression of putative target genes during cortical development^39,40^. Song et al. even demonstrated that 20–40% of active regulatory elements that interact with the promoters of protein-coding genes are specific to particular neuronal subtypes, underlining their importance in cell-type specific regulation^41^.

Enhancers can be located exonic, intronic, or intergenic. They can act upon their target promoter from up to megabases away, even skipping intervening genes. These distal enhancers are brought into close physical proximity with their target promoters via chromatin looping (Fig. 1)^42,43^. Enhancer–promoter (E–P) loop formation has been strongly associated with gene activation during neurodevelopment. Throughout mouse neural development, for example, dynamic E–P interactions are formed at the time of gene activation and disappear upon gene repression^44^. Also during lineage commitment of human embryonic stem cells into early neural precursors, the rewiring of E–P contacts happens in conjunction with changes in chromatin state and target gene expression^45^. Although chromatin looping has now been widely accepted as the predominant mechanism underlying E–P interaction, recently several cases have been described in which this mechanism does not apply and E–P distance even increases upon gene activation, suggesting alternative modes of E–P communication might be in play^46,47^.

Variants affecting enhancer elements have been linked to several NDDs, which are now part of a rapidly expanding class of what are sometimes collectively termed “enhanceropathies”^48^. For example, enhancers active during cortical neurogenesis and in different neural cell types are enriched for common variants associated with cognitive function and psychiatric disorders^41,49^. Moreover, de novo variants identified in patients with NDDs were also found to be enriched in regulatory elements, including promoter regions^50^ and brain-specific enhancer elements^51^ (Vas et al., unpublished data). For a more detailed discussion on enhancer function in brain development and disease we refer to other reviews^52–54^.

### 3D chromatin conformation

As discussed earlier in the context of E–P looping, gene regulation cannot be interpreted on a linear scale. Indeed, the human genome is organized into a hierarchical 3D structure (reviewed in^55^). On the smallest level E–P loops facilitate communication between enhancers and their target promoters^42,43^. E–P interactions are mostly confined to topologically associated domains (TADs), delimited by CTCF-bound insulator elements (Fig. 1). These insulated domains spatially constrict interactions, limiting E–P communication across their boundaries. TADs are thought to be formed through a “loop extrusion” mechanism, in which cohesin extrudes a chromatin loop through its ring-shaped structure until it runs into convergent CTCF-bound sites. At the highest level, compartments group active (A compartments) or inactive (B compartments) TADs. TADs switch compartments (i.e., compartment switching) when they become activated or repressed, for example during differentiation^56^.

The functional importance of this organization during neurodevelopment is highlighted by two studies demonstrating a massive rewiring of 3D genomic structures during mouse^44^ and human^57^ neural differentiation, including changes in compartmentalization, an increase in TAD size and interaction strength and the formation (or pruning) of dynamic chromatin loops. Rajarajan et al. found that these dynamic interactions also include SCZ-associated sequences^57^. There is indeed ample evidence that such structural changes play a role in the etiology of NDDs. For example, variants affecting the architectural proteins CTCF, YY1, and STAG1 (a cohesin subunit) were found to cause ID^58–60^, while the multisystem disorder Cornelia de Lange syndrome is frequently associated with variants in the cohesin loading factor NIPBL and the cohesin subunits SMC1A and SMC3^61^.

### Accessible tools for interpreting variation in the noncoding genome

The past decade, several consortia have made considerable efforts to comprehensively map (ncRNA) expression, regulatory elements, as well as 3D genomic interactions, across a variety of cell types, tissues, and developmental stages. These large data sets are typically easily accessible through dedicated websites or genome browsers and consulting them should always be considered as a first step in assessing the functional consequence of noncoding variants (Table 1). For example, Middelkamp et al. devised a computational pipeline, based on a combination of phenotype association and publicly available chromatin organization data, to predict driver genes that were directly or indirectly affected by SVs in patients with multiple congenital anomalies and ID^62^. However, many functional elements remain to be discovered and/or have not been experimentally validated. These lacunae in the functional annotation of the noncoding genome, as well as the lack of experimental validation, complicate the interpretation of noncoding variation. Therefore, additional functional assays might be required to fill the gaps. Recently, experimental strategies to identify and validate regulatory elements were extensively reviewed by Gasperini et al.^63^.

**Table 1:** Publicly available resources for the functional interpretation of noncoding variants.

Resource	Data type	Data sets	Brain tissues?	Reference
FANTOM	Expression profiling	CAGE	975 human tissues and cells, including multiple brain tissues and cell types	^122^
ENCODE	Expression profiling, epigenetic profiling	RNA-seq, ChIP-seq	Currently 46 brain tissues, cell lines and primary cells	^14^
ROADMAP	Expression profiling, epigenetic profiling	RNA-seq, ChIP-seq	111 human tissues and cell types, including 10 brain tissues	^123^
PsychENCODE	Expression profiling, epigenetic profiling, genomic interactions	(Single-cell) RNA-seq, ChIP-seq, ATAC-seq, Hi-C	60 postmortem human brains, ranging from embryonic to adult	^124^
3D Genome Browser	Genomic interactions	Hi-C, Capture Hi-C, ChIA-PET	Both human and mouse neural tissues and cells: cerebellum, cortex, hippocampus, neural progenitor cells	^96^
VISTA Enhancer Browser	Enhancer validation	Transgenic mice enhancer assays	Whole mouse embryos	^95^

## STRUCTURAL VARIATION IN NEURODEVELOPMENTAL DISORDERS

The genetic etiology underlying NDDs is heterogeneous, ranging from large chromosomal aberrations (SVs) to SNVs, affecting hundreds of genes^64^. Variants that have arisen de novo account for the majority of cases^65^. For example, ~60% of severe ID cases can be explained by de novo variants (both SNVs and SVs) in known ID genes^66^. However, rare inherited variation has also been shown to contribute to the pathogenesis of NDDs. Inherited SVs contribute in almost 4% of ASD cases^9^. In addition to these rare de novo and inherited variants, even common variants add to disease predisposition^67^.

### Noncoding regulatory SVs are enriched in NDDs

As labs are shifting to GS, there has been a tremendous increase in the identification of variants within noncoding DNA sequences, both in healthy and diseased individuals. For example, only ~0.5% of SNVs identified through GS lie within coding exons^1^. Interpreting the functional effect of these noncoding variants represents an enormous challenge. Most noncoding SNVs and even small indels, unless they disrupt a crucial functional site (e.g., TF binding site [TFBS]), have a low probability of affecting the function of regulatory elements. Indeed, it has been estimated that only 0.15% of possible SNVs in active brain enhancers could cause NDDs^51^. Even so, it is estimated that de novo SNVs in putative regulatory elements could explain up to 5% of NDD cases^6,51,68^.

SVs affecting noncoding regions, on the other hand, remove or multiply kilobases of DNA sequence or even relocate entire sections of chromosomes and are therefore more likely than SNVs to have a biologically meaningful impact on gene regulation. Redin et al. demonstrated that 7% of balanced translocations in NDDs cause the disruption of TADs^69^. Also, SVs affecting regulatory elements seem to be depleted in the normal population, underscoring their potential to be deleterious^9,70^. Brandler et al. showed SV depletion in *cis*-regulatory elements (transcription start sites [TSSs], 3’UTRs, and fetal brain promoters) of loss-of-function intolerant genes^9^, while Han et al. found SVs, especially those disrupting CTCF sites, to occur at significantly lower frequencies than intergenic SVs^70^.

Others have demonstrated a direct link between noncoding SVs and neurodevelopmental disease. De novo CNVs encompassing human accelerated regions with regulatory activity are implicated in up to 1.8% of ASD cases^71^. Also in the context of ASD, Turner et al. found a slight excess of de novo CNVs in putative regulatory elements within the vicinity of ASD genes, although this was not statistically significant due to a limited sample size^10^. Similarly, Monlong et al. detected an enrichment of noncoding CNVs near known epilepsy genes^72^, while Brandler et al. showed an enrichment of paternally inherited regulatory SVs in ASD cases^9^. Within the latter ASD cohort no de novo regulatory SVs were identified, illustrating another mode of NDD etiology and underscoring that screening of larger NDD cohorts will be required to uncover the full spectrum of noncoding SVs underlying NDDs.

### Functional consequence of noncoding SVs

The pathogenicity of SVs affecting protein-coding genes can in most cases be explained, quite intuitively, by gene dosage effects. However, the effect of noncoding aberrations on gene expression is more difficult to predict. Still, it has been shown that noncoding SVs alter the expression of nearby genes with larger effect sizes than noncoding SNVs or indels^73^. Moreover, the expression of target genes is negatively correlated with the total sum of enhancer sequence affected by an SV (both for deletions and duplications)^70^. Both observations demonstrate a link between the number of affected nucleotides and the magnitude of the functional effect.

There are several extensively studied cases that have given insight into the different mechanisms through which noncoding SVs can impact gene regulation, 3D chromatin structure, and, eventually, influence gene expression and phenotypic outcome (reviewed in detail in^12,43^). These include both dosage and positional effects (i.e., they can change either the copy number or order of regulatory elements). CNVs that delete, duplicate, or amplify noncoding sequences potentially alter the dosage of ncRNA genes or regulatory elements, in turn affecting the expression of target genes. However, this effect on target gene expression might be difficult to predict. As gene expression is often the result of the combinatorial action of multiple enhancer elements with (partly) overlapping activity patterns, the degree of enhancer redundancy will influence the functional consequences. For example, the individual deletion of ten different enhancer elements at loci involved in limb development did not result in a limb phenotype, while the deletion of pairs of enhancers did^38^.

SVs that span TAD boundaries can, in addition to causing dosage effects, also influence 3D chromatin structure. The deletion of a TAD boundary facilitates the fusion of adjacent TADs, while a duplication involving a TAD boundary can result in the creation of a new chromatin domain or neo-TAD. Inversions and translocations reshuffle the TAD structure by repositioning boundaries. These alterations in the 3D chromatin structure can bring about the decoupling of a promoter from its cognate enhancers resulting in a regulatory loss of function, while at the same time the adoption of new enhancers with different spatiotemporal activities might lead to ectopic gene activation^74^. However, these mechanisms are not necessarily generalizable. For some loci the removal of TAD boundaries and ensuing TAD fusion does not appear to result in gene expression changes^75^.

## SVs IMPACTING GENE REGULATION IN NDDs: SPECIFIC CASES

Because of the complexity of the gene regulatory landscape illustrated above, the medical interpretation of SVs in noncoding regions requires a case-by-case evaluation of the functional impact on gene expression and phenotypic outcome. To better understand the diverse ways in which noncoding SVs contribute to the etiology of NDDs, we have compiled an extensive list of loci harboring putatively causal, noncoding SVs in patients with NDDs (Table 2).

**Table 2:** Examples of noncoding structural variants (SVs) putatively causal in neurodevelopmental disorders (NDDs).

Locus	Affected gene^a^	SV type	Noncoding disease mechanism	NDD phenotype	Reference
lncRNAs					
22q11.2	*DGCR5*	Translocation, deletion	*DGCR5* haploinsufficiency	DiGeorge syndrome; SCZ	^76,77^
5q15	*lnc-NR2F1*	Translocation, deletion	*lnc-NR2F1* haploinsufficiency	DD, facial dysmorphisms, hearing loss	^78^
Xp22.11	*PTCHD1-AS*	Deletion	*PTCHD1-AS* haploinsufficiency	ASD	^79^
14q21.1	*lncLRFN5-10*	Deletion	*LRFN5* haploinsufficiency	ASD	^80^
2p25.1	*LINC00299*	Translocation, deletion	*LINC00299* haploinsufficiency	DD, ID	^81,82^
12q23.1	*RMST*	Translocation	*RMST* haploinsufficiency	Kallmann syndrome	^85^
14q32.2	*MEG3*	Deletion (maternal)	*MEG* haploinsufficiency	upd(14)pat phenotype	^86^
15q11-13	*SNORD116*	Deletion	snoRNA haploinsufficiency	Prader–Willi syndrome	^87,88^
Near *cis*-regulatory elements (promoter and 5’&3’ UTR)					
Xq27.3	*FMR1*	CGG repeat expansion	Promoter hypermethylation	Fragile X syndrome	^90^
16p12.3	*XYLT1*	GGC repeat expansion	Exon 1 hypermethylation	Baratela–Scott syndrome	^92^
Xq28	*AFF2*	CCG repeat expansion	Promoter hypermethylation	ID	^90^
2q11.2	*AFF3*	CGG repeat expansion	Promoter hypermethylation	ID	^90^
12q13.1	*DIP2B*	CGG repeat expansion	Promoter hypermethylation	ID	^90^
16q21	*GPR56*	Deletion	Promoter TFBS disruption	Polymicrogyria, ID, speech delay, seizures	^52^
Intergenic regulatory elements and 3D chromatin conformation					
7q36.3	*SHH*	Translocation	Enhancer displacement	Holoprosencephaly	^93^
14q12	*FOXG1*	Translocation, deletion	Enhancer displacement/removal or 3D reorganization	Congenital Rett syndrome	^69,97^
5q14.3	*MEF2C*	Translocation, deletion, inversion	Enhancer displacement/removal	Rett-like syndrome	^69,98^
2q33.1	*SATB2*	Translocation, inversion	Enhancer displacement	Glass syndrome	^69,100^
17q24.3	*SOX9*	Translocation, deletion duplication	Enhancer displacement/removal, Enhancer adoption	Pierre Robin sequence Cooks syndrome, sex reversal	^101,102^
Xq27.1	*SOX3*	Insertion, deletion	Enhancer insertion/removal	Variable phenotypes	^62,104,105^
7q21.3	*DLX5/6*	Translocation, deletion, inversion	Enhancer displacement/removal	SHFM1, ID, craniofacial defects, hearing loss	^107,108^
4q25	*PITX2*	Translocation, deletion	Enhancer displacement/removal	Rieger syndrome	^69,111,112^
11p13	*PAX6*	Translocation	Enhancer displacement	Aniridia	^52,113^
Xp21.3	*ARX*	Duplication	Enhancer duplication	ID, epilepsy, lissencephaly	^52^
6p24.3	*TFAP2A*	Translocation, inversion	Enhancer displacement, 3D reorganization	BOFS	^114^
14q32.2	*BCL11B*	Translocation	Enhancer removal	DD, speech delay, ID	^115^
1p34.2	*SLC2A1*	Translocation	Enhancer displacement	Epilepsy, DD	^69^
7q36.3	*VIPR2*	Duplication	Unknown	SCZ	^52^
15q26.2	*NR2F2*	Duplication	Enhancer duplication	ASD, ID	^71^
21q22.2	*DSCAM*	Intronic deletion	Enhancer removal	ASD	^10^
2p12	*CTNNA2*	Translocation	Enhancer displacement	ID, DD	^116^

*ASD* autism spectrum disorder, *BOFS* branchiooculofacial syndrome, *DD* developmental delay, *ID* intellectual disability, *SCZ* schizophrenia, *SHFM1* split hand/foot malformation 1, *TF* transcription factor, *TFBS* TF binding site.

^a^Directly or indirectly.

### lncRNAs disrupted by SVs in NDDs

Although differential expression of lncRNAs has frequently been linked to NDDs^29–31^, it is often difficult to disentangle association from causation. Cases in which disruption of a specific lncRNA gene appears to be the causal disease mechanism are still limited in number and come with a varying degree of functional evidence. Therefore, it remains unsure whether this is a widespread phenomenon in the etiology of NDDs and disease in general.

By 1996, chromosomal aberrations affecting the lncRNA gene *DGCR5* had been identified in patients with DiGeorge syndrome^76^. More recently, CNVs situated within the same 22q11.2 critical region were found to be associated with SCZ as well^7^. Although this region also harbors several protein-coding genes, based on coexpression analysis using brain transcriptome data from the PsychENCODE project, *DGCR5* was identified as a hub regulator^77^. Moreover, *DGCR5* knockdown and overexpression in induced pluripotent stem cell (iPSC)–derived human neural progenitors demonstrated that this lncRNA regulates the expression of several SCZ-associated genes^77^.

**Fig. 2:**
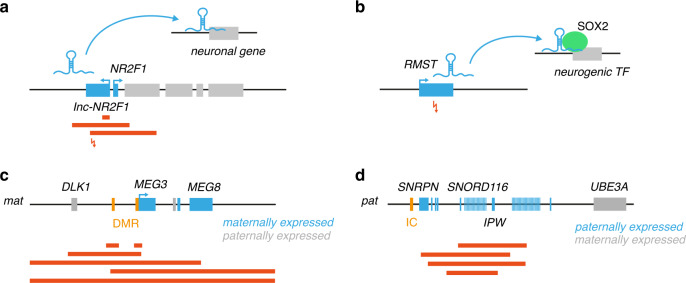
Structural variants disrupt noncoding RNA genes in patients with neurodevelopmental disorders (NDDs). Illustration of NDD cases in which the disruption of a noncoding RNA locus has been identified as the causal mechanism (four examples, **a**–**d**). (Noncoding) genes are depicted as blue and gray boxes, red bars are patient deletions, arrows are translocation breakpoints. The epigenetically regulated MEG3 differentially methylated regions (DMRs) and Prader–Willi imprinting control (IC) region are represented by orange boxes.

Very recently, differential expression analysis during mouse neuronal induction implicated the highly conserved *lnc-NR2F1*, transcribed divergently from the *NR2F1* locus, in neurodevelopment^78^. Overlap of this lncRNA with a focal deletion found in ASD/ID patients and a newly identified, paternally inherited translocation confirmed its clinical relevance in NDD etiology (Fig. 2a). Both gain- and loss-of-function experiments in mouse have demonstrated that *lnc-Nr2f1* promotes neuronal maturation pathways in a functionally distinct fashion from its neighboring gene *Nr2f1*, while chromatin association assays showed that it binds to neuronal targets in *trans* to exert that function^78^. Also in the context of ASD, *PTCHD1-AS* was found to be frequently affected by microdeletions in male patients^79^. This lncRNA lies upstream of the *PTCHD1* gene, which is a transmembrane protein known to be involved in NDDs. iPSC-derived neurons of patients with *PTCHD1-AS* deletions showed decreased excitatory synaptic activity, although PTCHD1 expression does not appear to be affected. This in contrast to *lncLRFN5-10*, which does appear to regulate expression of its nearby gene *LRFN5* and was also found to be affected by a microdeletion in an ASD patient^80^. Translocations and deletions disrupting *LINC00299* have been identified in patients with DD and ID^81,82^. Although it has been demonstrated that *LINC00299* expression increases during neural differentiation, its precise function is still unknown.

*RMST* was also first associated with neurodevelopment through transcriptomic analyses. This lncRNA was found to be upregulated during neuronal differentiation and was shown to regulate this process through interaction with the SOX2 TF^83,84^. Recently, a de novo balanced translocation disrupting *RMST* was identified in a patient with Kallmann syndrome, a disorder caused by deficient development of gonadotropin-releasing hormone (GnRH) neurons and featured by abnormal sexual development and an impaired sense of smell (Fig. 2b)^85^. The translocation caused a reduction in *RMST* expression in patient neural crest cells (NCCs), the cells from which GnRH neurons originate, resulting in abnormal NCC morphological development^85^.

The dysregulation of lncRNAs within imprinted regions has also been associated with NDDs. For instance, microdeletions involving the differentially methylated regions (DMRs) of maternal origin upstream of the maternally expressed gene 3 (*MEG3*) lncRNA result in a loss of *MEG* expression and a phenotype resembling that of paternal uniparental disomy 14 (upd[14]pat) patients (growth retardation, DD, facial abnormalities, small bell-shaped thorax, abdominal defects, and polyhydramnios) (Fig. 2c)^86^. Finally, the deletion of the imprinted *SNORD116* noncoding gene cluster on the paternal allele results in Prader–Willi syndrome (PWS) (Fig. 2d)^87,88^. *SNORD116* is processed into 30 small nucleolar RNAs (snoRNAs). The production of these snoRNAs appears to be neurospecific^89^ and their deletion results in smaller neuronal cell bodies due to a decrease in nucleolar size^88^. However, the precise role of these snoRNAs in the nucleolus remains unsolved.

### Disruption of near *cis*-regulatory elements (promoter, 5’ & 3’ UTR) in NDDs

As near *cis*-regulatory elements, such as the promoter and 5’ and 3’ UTR, are typically small in size and directly flanking the coding sequence, they are inherently less likely to be affected by SVs that leave the protein-coding gene body intact. However, there are a few examples of larger genomic variants disrupting these near-*cis* sequences in the context of NDDs. Undoubtedly one of the most well-known examples is the CGG repeat expansion in the 5’ UTR of the *FMR1* gene, resulting in DNA hypermethylation at the promoter, silencing *FMR1* and giving rise to fragile X syndrome^90^. Interestingly, this repeat expansion also disrupts the TAD boundary adjacent to *FMR1*, decoupling *FMR1* from putative downstream enhancers^91^. As the extent of the disruption correlates to *FMR1* silencing, it is possible that part of the FMR1 loss is attributable to this 3D rearrangement. Repeat expansions in near *cis*-regulatory elements are a recurrent cause of NDDs. A GGC repeat expansion in the *XYLT1* promoter of patients with Baratela–Scott syndrome results in hypermethylation of the first exon and reduced XYLT1 expression^92^. Repeat expansions also give rise to other forms of ID, including FRAXE (5’ UTR of *AFF2*), FRA2A (promoter of *AFF3*), and FRA12A (5’ UTR of *DIP2B*)^90^.

Other types of SVs have also been reported within near *cis*-regulatory sequences. For example, a deletion in the promoter region of *GPR56*, disrupting an RFX TFBS, leads to gyral malformations in a specific region of the cortex, resulting in speech delay, ID, and seizures^52^. Variants affecting the coding sequence of *GPR56* typically result in polymicrogyria of the entire cortex. However, as the regulatory deletion is located within the promoter of only one of multiple alternative TSSs, it only eliminates GPR56 expression in the lateral neocortex, explaining the regionally restricted phenotype.

### SVs disrupt long-range gene regulation and 3D chromatin structure in NDDs

**Fig. 3:**
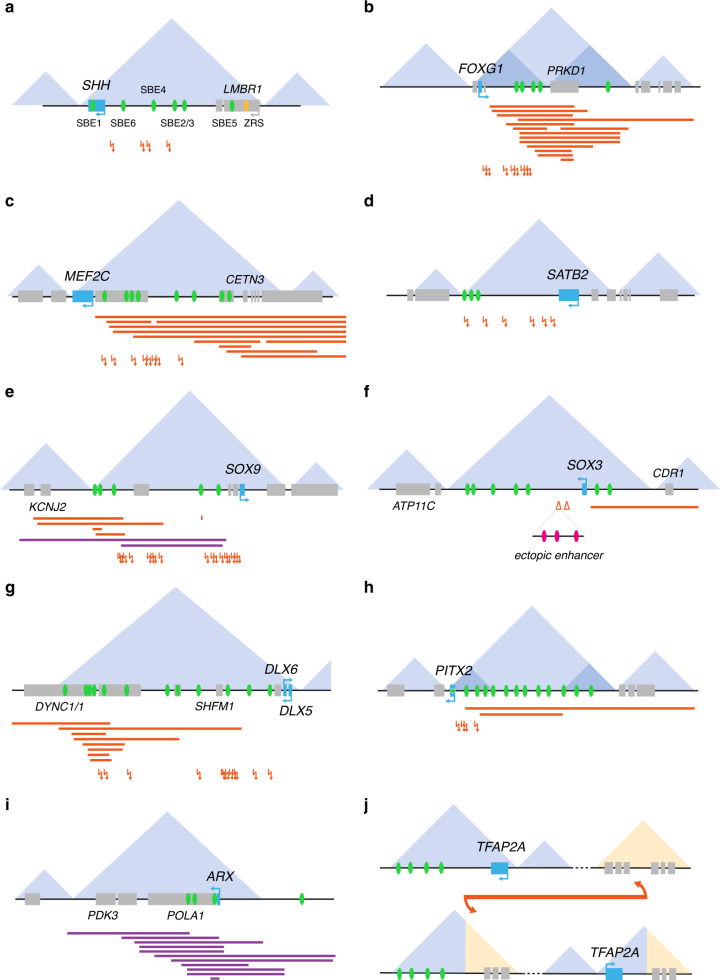
Noncoding structural variants disrupt gene regulation and 3D chromatin structure in neurodevelopmental disorders (NDDs). Illustration of NDD cases in which the disruption of regulatory elements and/or 3D chromatin conformation has been identified as the causal mechanism (ten examples, **a**–**j**). Blue triangles reflect the topologically associated domain (TAD) structure of the locus. Depicted are (noncoding) genes represented by blue (gene of interest) and gray (other gene) boxes, brain enhancer elements (green ovals), patient deletions (red bars), duplications (purple bars), insertions (red triangles), and translocation or inversion breakpoints (red arrows).

Most noncoding SVs associated with NDDs are situated within the large stretches of intergenic space, affecting regulatory interactions between promoters and enhancers and/or altering the 3D chromatin conformation of the locus (see section above). Variants affecting the ZRS limb enhancer at the *SHH* locus are well known to cause limb malformations. However, translocations upstream of *SHH*, disrupting the interaction between the *SHH* promoter and *SHH* brain enhancers (SBE6, SBE4, SBE2, and SBE3), have been identified as a cause of holoprosencephaly (Fig. 3a)^93,94^.

The FOXG1 TF has been associated with a congenital form of Rett syndrome, an NDD featured by severe DD, absence of speech, seizures, hypotonia, and stereotypic movements. The gene is located in a large, gene-poor TAD with a pronounced sub-TAD structure and multiple interaction loops, bringing the promoter into the proximity of several in vivo validated brain enhancers^95,96^. This regulatory structure is disrupted by translocations and deletions distal to *FOXG1* in multiple patients with similar Rett-like features (Fig. 3b), likely caused by either enhancer deletion/translocation or a rewiring of interactions due to the deletion of TAD boundary elements^69,97^. SVs in the 5q14.3 region upstream of *MEF2C* result in a Rett-like syndrome as well (Fig. 3c)^69,98^. This upstream region harbors multiple enhancer elements that display in vivo neuronal activity during zebrafish development and form a physical interaction network with the *MEF2C* promoter in neuronal cells, suggesting that *MEF2C* transcriptional dysregulation by enhancer deletion or translocation lies at the root of the MEF2C-related phenotype^98^.

Loss-of-function variants in SATB2 or microdeletions in the 2q33.1 region affecting *SATB2*, typically give rise to SATB2-associated syndrome, an NDD characterized by ID and dysmorphic facial features^99^. Six patients with BCA breakpoints in the gene desert 3’ to *SATB2* exhibited overlapping clinical features (Fig. 3d)^69,100^. Each of these breakpoints disrupts the long-range interactions between *SATB2* and multiple putative enhancer elements, of which at least one (CRE2) drives *SATB2*-like craniofacial expression in zebrafish^100^. Interestingly, the activity of this element appears to be dependent on binding of the SOX9 TF, which has been associated with a craniofacial disorder with overlapping clinical features called Pierre Robin sequence (PRS), suggesting that *SATB2* regulation might be primarily driven by SOX9. Translocations and microdeletions upstream of *SOX9* have also been identified in patients with PRS, while duplications involving the TAD boundary give rise to Cooks syndrome and intra-TAD duplications cause sex reversal (Fig. 3e)^101,102^. The effects of these SVs on gene regulation and 3D conformation at the *SOX9* locus have been discussed at length by others^12,103^. Duplications and deletions of the SOX3 TF cause ID and growth hormone deficiency. Intriguingly, multiple SVs have been identified in the gene desert surrounding *SOX3* in patients with varying phenotypes without ID (Fig. 3f). A region 82 kb downstream of the gene is especially prone to insertions due to the presence of a human-specific short palindromic sequence. The distinct symptoms observed in these patients suggest the phenotypes might be caused by the introduction of tissue-specific enhancer elements driving ectopic SOX3 expression. A 170-kb fragment from chromosome 9 was inserted at this site in a patient with cleft palate and facial dysmorphism^62^. The insertion contained part of a superenhancer region with craniofacial activity, possibly altering SOX3 expression during craniofacial development and resulting in the patient’s phenotype. Insertions of other genomic fragments resulted in a severe hair overgrowth phenotype (hypertrichosis), drooping eyelids (ptosis), XX male sex reversal, and X-linked recessive hypoparathyroidism^104,105^. Additionally, an upstream microdeletion was identified in a patient with XX male sex reversal^106^.

Chromosomal aberrations affecting the *DLX5/6* locus cause split hand/foot malformation 1 (SHFM1), often combined with ID, craniofacial defects, and hearing loss. Several of these SVs have breakpoints upstream of *DLX5/6* and disrupt multiple tissue-specific enhancer elements, which regulate DLX5/6 expression in the forebrain, branchial arch, ear, and limb (Fig. 3g)^107,108^. Patients can even be classified into three phenotypic groups, correlating with the deletion of specific enhancer elements^107–109^. Variants disrupting the PITX2 TF are typically associated with Rieger syndrome, a developmental disorder characterized by ocular and craniofacial anomalies, with some patients also displaying neurological deficits^110^. Translocations and deletions affecting conserved enhancer elements (with brain, eye, and craniofacial activity) in the gene desert upstream of *PITX2* result in a similar phenotype (Fig. 3h)^69,111,112^. Variants in the coding sequence of *PAX6* give rise to the congenital eye malformation aniridia, as well as neurodevelopmental defects. Translocations in the downstream regulatory region were found to result in a similar phenotype^52,113^.

While many of these examples consist of deletions or translocations, duplications of noncoding sequences have also been associated with NDDs. Coding variants or small duplications in the ARX TF are a frequent cause of X-linked ID, epilepsy, and lissencephaly. Its specific expression in different regions of the forebrain is tightly controlled by ultraconserved enhancers downstream of the coding sequence, with partially overlapping spatial activity patterns^39^. Duplications encompassing these *ARX* forebrain enhancers cause a similar, although milder, phenotype (Fig. 3i)^52^.

Also in patients with complex genomic rearrangements noncoding SVs can contribute to the overall phenotype. For example, Middelkamp et al. identified four candidate driver genes (i.e., *PHIP*, *COL12A1*, *BMP2*, and *TFAP2*) in a patient with a complex rearrangement consisting of six breakpoint junctions and two deletions on three different chromosomes^62^. Each of the driver genes individually can only account for part of the phenotype (i.e., DD, autism, seizures, facial dysmorphism, growth delay, missing ribs, renal agenesis, and cryptorchidism), yet together they might explain the full phenotypic spectrum. *PHIP* and *COL12A1* were directly affected by a deletion and have been associated with DD and facial dysmorphisms. In addition, *BMP2* and *TFAP2A* appeared to be affected by a disruption of long-range interactions. Several breakpoints were identified upstream of *BMP2*, linked to short stature, facial dysmorphisms, and skeletal anomalies, and also the *TFAP2A* TAD was disrupted by a translocation. Recently, a de novo heterozygous inversion disrupting the *TFAP2A* TAD was also identified in a patient with branchiooculofacial syndrome (BOFS; branchial cleft, ocular anomalies, facial dysmorphisms) (Fig. 3j)^114^. The TFAP2A TF regulates neural crest development and its expression in NCCs is controlled by multiple enhancers. Laugsch et al. have demonstrated that the inversion separates *TFAP2A* from its NCC enhancers, leading to monoallelic expression and TFAP2A haploinsufficiency. Interestingly, no enhancer adoption occurs in this case, even though the inversion places these relocated enhancers within the spatial proximity of other genes.

Finally, in some cases functional evidence for a noncoding disease mechanism is still limited. For example, translocations 3’ to the *BCL11B* TF gene were found in patients with DD, speech impairment, and ID^115^. Expression was reduced by 50% in patient cells, suggesting BCL11B haploinsufficiency due to relocation of regulatory elements. Redin et al. identified a patient, displaying epilepsy and DD, with a translocation affecting the *SLC2A1* TAD and decreasing SLC2A1 expression in patient cells^69^. The translocation disrupts the interaction between *SLC2A1*, a gene associated with the seizure disorder GLUT1 deficiency syndrome, and several putative enhancer elements. Duplications of the 7q36 region, either including or just upstream of *VIPR2*, result in upregulation of VIPR2 and cause schizophrenia in patients^52^. Interestingly, it seems like the overexpression pattern cannot only be explained by an increase in gene dosage, suggesting that these duplications affect *VIPR2* regulation as well. A de novo duplication 300 kb upstream of *NR2F2*, a gene associated with ASD and ID, duplicates a human accelerated region that has been shown to interact with the *NR2F2* promoter, possibly exerting a regulatory function during neural development^71^. Intronic CNVs can also affect gene regulation. A 14-kb inherited, intronic deletion in the *DSCAM* gene has been identified in an autism patient^10^. The deletion removes at least nine enhancer elements driving expression in the central nervous system (CNS). Recently, Melo et al. identified a translocation disrupting the *CTNNA2* TAD in a patient with ID and DD^116^. Although homozygous variants in this gene cause cortical dysplasia and other brain malformations, a noncoding disease mechanism has not yet been investigated. Furthermore, putatively disease-causing SVs have been found disrupting the noncoding regions surrounding *RAP1A* (Kabuki syndrome), *PPP3CA* (epilepsy and ID), *RAC1* (ID), *PAFAH1B1* (lissencephaly), *ALX4* (Potocki–Shaffer syndrome), *FOXP2* (speech and language disorder), and *TGFB2* (~ID)^62,117^.

## CONCLUSION AND PERSPECTIVES

The cases discussed above clearly demonstrate the importance of considering noncoding effects when interpreting SVs in the context of NDDs. Studying these cases and experimentally investigating gene regulation within these loci could greatly improve our understanding of the noncoding disease mechanisms at play, ultimately benefiting the medical interpretation of structural variation. For many, however, the underlying noncoding disease mechanism has not yet been fully resolved. This is especially true for the SVs putatively disturbing long-range gene regulation and/or 3D chromatin structure. In many of these cases a disruption of communication between the promoter and its cognate enhancer sequences, either due to enhancer deletion or relocation, is thought to be the causal mechanism. However, (part of) the effect may also be caused by the acquisition of new interactions as a result of TAD fusion or reshuffling. Further experimental validation will be needed to ascertain how these different mechanisms contribute to the disease phenotype.

There are some loci for which the effect of noncoding SVs has been extensively studied, especially in the context of limb malformations^12^. These studies have demonstrated that the effects are locus-dependent and are therefore difficult to predict. This clearly exemplifies the complexity of the gene regulatory landscape and our imperfect understanding of the role, determinants, and necessity of the 3D chromatin structure. Also hampering the functional assessment of structural variation is the incomplete annotation of the noncoding genome on a tissue-specific level. Although several large studies have predicted the presence of functional elements throughout the genome for a variety of tissues and cell types, these predictions are based on biochemical properties (e.g., TF binding, open chromatin, histone modifications) and do not guarantee that these sequences perform a regulatory function in vivo. As a result, most of these putative functional elements still require experimental validation, be it via high-throughput screening assays (e.g., ChIP/ATAC-STARR-seq^118,119^ or CRISPR(i) screening^120^) or in focused studies.

A more complete annotation of the noncoding genome and a better understanding of different noncoding disease mechanisms will also improve our ability to predict the transcriptional and phenotypic consequences of newly identified, noncoding SVs. This is not only true for the large de novo SVs discussed here, but also for (combinations of) inherited variants with smaller individual effects. Moreover, while this review focused on structural variation, an enormous challenge is looming ahead to interpret the millions of noncoding SNVs identified in patient genomes as well. In contrast to the relatively large SVs, most of these single-nucleotide changes are likely to have no (or very small) functional effect(s), rendering the prediction of variant effects, prioritization, and validation possibly even more crucial.

A multiomics approach has been proposed to unify variant detection and interpretation, by combining information on a genomic, epigenomic, transcriptomic, and even functional genomic level^121^. This could be achieved, for example, by simultaneously implementing GS for variant identification, Hi-C analysis of 3D chromatin structure, RNA-seq profiling of transcriptional activity, and high-throughput assays for the functional validation of putative regulatory elements. Such an approach should ultimately aid in closing the gap in the genetic diagnosis of NDD patients, while at the same time improving our understanding of gene regulatory mechanisms.
